# Missed opportunities to promote flourishing in cancer care: a brief examination of multiple myeloma

**DOI:** 10.1007/s00520-026-10978-3

**Published:** 2026-07-08

**Authors:** Natalie Tuckey, Hannah R. Wardill, Xavier Symons, Melissa Cantley, Kathina Ali, Hayley Beer, Gregory B. Crawford, Angelina Yong, Matthew Iasiello

**Affiliations:** 1 Supportive Oncology Research Group, Precision Cancer Medicine Theme, SAHMRI, Adelaide, SA Australia; 2 School of Pharmacy and Biomedical Science, College of Health, Adelaide University, Adelaide, SA Australia; 3 Human Flourishing Program in the Institute for Quantitative Social Science, Harvard University, Cambridge, MA USA; 4 Plunkett Centre for Ethics, The Australian Catholic University, Sydney, NSW Australia; 5 Precision Cancer Medicine Theme, SAHMRI, Adelaide, SA Australia; 6 Myeloma Research Laboratory, School of Pharmacy and Biomedical Science, College of Health, Adelaide University, Adelaide, SA Australia; 7 School of Health, University of the Sunshine Coast, Sunshine Coast, QLD Australia; 8 Myeloma Australia, Parramatta, NSW Australia; 9 School of Medicine, College of Health, Adelaide University, Adelaide, SA Australia; 10 Central Adelaide Local Heath Network, SA Health, Adelaide, SA Australia; 11 Be Well Co, North Sydney, NSW Australia

**Keywords:** Cancer, Myeloma, Treatment, Psychosocial, Flourishing, Mental health, Smouldering myeloma, End of life, Death and dying

## Abstract

Recent theoretical and empirical advancements in well-being science can have meaningful implications for medical care when considering existential issues faced by people with incurable or life-limiting cancer diagnoses. We propose that certain elements of flourishing, such as meaning and purpose, deep personal relationships, and cultivating virtue, can be uniquely realized if targeted appropriately toward the end of life. Qualitative thematic analysis of semistructured interviews was conducted to explore the experiences and unmet psychological needs of people living with the blood cancer multiple myeloma and, precursor, smoldering myeloma to identify key themes related to the potential for flourishing at the end of life. Interview data was obtained from 25 participants (multiple myeloma *n* = 24, smoldering myeloma *n* = 4) and 10 health professionals with clinical expertise supporting or treating patients with multiple myeloma. Participants reported personal growth following their diagnosis through acceptance, meaning-making, and connection, yet reported the loneliness of dying with cancer with a desire to have discussions about death with their clinicians in addition to them maintaining treatment. On the contrary, health professionals were hesitant to discuss death with their patients, balancing hope in treatments with preparation for dying. We argue that the hesitation to discuss death thwarts the opportunity to flourish and that existing solutions such as dignity therapy, meaning-centered psychotherapy, early palliative care, and multidisciplinary support can close this gap without diminishing hope in treatments.

**Supplementary Information:**

The online version contains supplementary material available at 10.1007/s00520-026-10978-3.

## Introduction

Well-being science has long been criticized for its predominant focus on the “worried well” [1]. However, progress in the field has identified key psychological predictors of physical illness, identifying psychological interventions that are effective in clinical care and can assist in recovery from mental and physical illness [2, 3]. A recent theoretical framework developed by Symons et al. [4] posits that flourishing—encompassing emotional, psychological, and social well-being—remains achievable *even* toward the end of life. This perspective challenges traditional views of terminal illness, suggesting that patients can experience and *build* meaning, purpose, and connection despite their prognosis [5]. This framework emphasizes holistic well-being, integrating concepts such as personal growth, relationships, and spiritual fulfillment. Symons et al. [4] argue that prevailing medical models, which prioritize physical health and symptom management, often neglect the broader dimensions of human experience critical to a good life. Similarly, Donaldson [6] offers a framework for a “good death” in patients with hematologic malignancies which integrates the notion of eudaimonia, and which emphasizes living well and dying well. The argument provides ethical guidance for balancing life-extending treatments with the promotion of a peaceful death [6]. These advancements offer the theoretical opportunity to *intentionally* support patients to flourish with life-limiting or end of life cancer diagnoses.

In partnership with lived experience experts, our team has launched the *myWELL* Study [7] working to modify, through co-design, an existing psychological intervention to support the unmet psychological needs of people with multiple myeloma (MM). MM is a type of blood cancer that currently has no cure and is associated with high rates of clinically significant anxiety and depression [8], related to the uncertainty of treatment outcomes, inevitability of relapse, and ultimately facing one’s own mortality. Therefore, it is a unique setting to explore the concept of flourishing at end of life, as the condition is manageable yet remains a terminal diagnosis, with enormous variation in survival rates and often a requirement to adhere to lifelong therapy [9]. In the current study, we aim to explore the experiences and unmet psychological needs of people living with MM and the precursor condition, smoldering myeloma (SMM), to identify themes related to the potential for flourishing.

## Methods

### Study design and setting

This qualitative study was embedded within the pilot of *myWELL* Study, a 5-week mental health and well-being intervention [7]. The methodology is summarized here to address the aim of this study.

### Ethics approval

Ethical approval was obtained from The Adelaide University Human Research Ethics Committee (H-2024-205).

### Participants and recruitment

#### Patients

Eligible participants were adults (aged 18 years or older) with a self-reported diagnosis of MM or SMM, who were fluent in English, and residing in Australia. Participants were recruited through a newsletter and targeted email from peak national body, Myeloma Australia. Participants completed an online expression of interest including general demographic questions (age, gender, location, time since diagnosed) and provided written informed consent to take part in the study. Participants were then contacted by telephone and invited to attend an onboarding interview. Participants were financially compensated for their time, in accordance with institutional guidelines [10].

#### Health professionals

Health professionals were eligible if they had current clinical experience supporting patients with MM and SMM in Australia. Purposeful sampling was used in order to acquire a wide range of views of supportive care available to myeloma patients. We approached professionals involved in the care and support of people living with MM and SMM, including hematologists, nurses, allied health professionals, and representatives from national myeloma and cancer support organizations. Participants were invited by email, with one follow-up reminder provided.

### Interview design

The semistructured interviews were based on the Supportive Cancer Care Framework [11] and not specific to death and dying. Interviews continued until the dataset was considered sufficiently rich and diverse to support meaningful thematic analysis and address the research questions [12]. The questions were initially shared with a hematologist, representative from Myeloma Australia, and experts in culturally and linguistically diverse (CALD) populations for inclusivity and appropriateness. The questions were then piloted with patient advocates (MM *n* = 1, SMM *n* = 1) to ensure appropriateness. The guide was used flexibly to guide discussions, which were subsequently transcribed verbatim and checked, corrected, and pseudonymized by the interviewer. The questions focused on the patient’s experiences with MM and SMM, their perspective on the care they receive for their physical and mental health, and preferences for supportive care (see supplementary information for interview questions)*.*

### Data collection

All interviews were conducted via Zoom by the first author, N.T., between November 2024 and March 2025, ranging from 19 to 54 min. They were audio-and video-recorded with consent.

### Data analysis

Reflexive thematic analysis framework was used as described by Braun and Clarke [13] involving the process of developing familiarity with the data, generating initial codes, searching for themes relevant to the research aims, reviewing themes, and reporting the findings. Led by N.T., supported by members of the research team, themes and subthemes were defined and refined iteratively through multiple consensus discussions.

## Results

Twenty-five patients participated in the semistructured interviews (MM *n* = 21, SMM *n* = 4). Patient demographic and clinical characteristics are summarized in Table 1. A total of 10 health professionals were interviewed (hematologist *n* = 3, myeloma nurses *n* = 3, GP *n* = 1, psychologist *n* = 1, physiotherapist *n* = 1, and social worker *n* = 1) who had experience across both private and public health settings.

**Table 1:** Patient characteristics

Characteristics	Multiple myeloma (*n* = 21)	Smoldering myeloma (*n* = 4)
Gender		
Male	10	2
Female	11	2
Age, mean (SD)	61.4 (10.5)	54.5 (4.7)
Location of residence		
Metropolitan	14	3
Rural/regional	8	1
Time since diagnosis		
Less than 2 years	5	2
Between 2 and 4 years	11	2
5 years or longer	5	0

Three themes were identified from the qualitative interviews: (i) growth despite a life-limiting diagnosis, (ii) the lonely burden of dying, and (iii) clinician reluctance to talk about death. Extended description of themes with illustrative quotes is summarized in Table 2.

**Table 2:** Qualitative themes and illustrative quotes from interviews with people living with myeloma, smoldering myeloma and health professionals

Theme	Subtheme	Illustrative quote
Growth despite a life-limiting diagnosis The theme explores growth in the final stages of life, focusing on acceptance of the inevitable, personal reflection, spiritual exploration, and the revaluation of what is meaningful	Acceptance of the inevitable	“Everyone’s going to die. And since I’ve been diagnosed in 5 years, I’ve lost people who haven’t had cancer, you know what I mean, and I’m dead quicker than me. So you can’t dwell. Walk out and get hit by a bus. You can’t dwell on life. It’s an infinite, you know. It’s finite, but you can’t tell when it’s going to end. So it’s a get out life is for living is the saying.” *(Male, MM, 60–70 years)*
	Personal growth and reflection	“So, yeah, so that’s kind of, in a way, it’s, you know, the sort of the parable of life is that you know, we are on this journey, and it’s different at different stages. And you learn from it. You know you, if you listen.” *(Male, 70–80 years)*
	Spirituality and dying	“What I would call spiritual is the work that I do, that’s like the meditation and breath work which is all about just and I mean, yeah, it’s that’s a longer conversation. But my mum just passed away and had the same woman helping her sort of through her death. And the whole concept was basically that you are just energy, and your energy takes a physical form. And then it doesn’t. And that’s kind of okay. And you can let that go.” *(Female, MM, 40–50 years)*
	Valuing what is important	“I just keep doing what I normally did in the past, and I’ve been building a train set for my grandson which is keeping away from screens and things, and we’re always in the shed doing things together. That keeps me occupied. It keeps me grounded.” *(Male, MM, 70–80 years)*
The lonely burden of dying The theme explores the personal challenges of facing death, including managing practical end-of-life matters, navigating the discomfort of others who avoid the topic	Practically preparing for death	“I’m a bit paralysed around the space of sorting will out because I feel like I’m going to have to have a will number one and will number two, you know, so even in that space, I’m not. I’m not prepared at all at the moment.” *(Female, SMM, 50–60 years)*
	Friends do not want to talk about death	“I’ve spoken to friends so much about it. I’ve found that most people are very scared to talk death, very scared to talk terminal illness. And ghosting was probably the worst thing I had during the whole lot of being diagnosed, and even to this day. People just don’t want to face death, and I think, that’s the main crux of it.” *(Male, SMM, 60–70 years)*
	Placating others for their benefit	“So you find that you’re actually placating other people. If that makes sense, trying to make them feel better. And rather than just say, look, you know, it is what it is. It’s cancer. It’s a word. I’m not afraid of it. But thank you.” *(Female, MM, 60–70 years)*
Clinician reluctance to talk about death Clinicians raise the discomfort of talking about death and the challenges for patients and families	Clinician discomfort and avoidance	“You don’t want to, I see that happen, the clinician doesn’t want to necessarily rehash that every time, because that patient’s at a critical point where they have to have hope. Otherwise, why would you wanting be wanting to sign up for a clinical trial. Why would you be coming in each week? And some people will want to fight until the very, very end.” *(Myeloma Nurse)*
	Concerns about impact on patients and families	“It’s often difficult to predict how the end of their life is going to progress, and I don’t think it’s particularly help. I don’t think it’s particularly helpful, you know. I have discussions when they’re approaching that about, you know. Where would they want to pass away? And you know what things are important to them. But I think talking about the actual process isn’t particularly. I think it’s distressing.” *(Hematologist)*
	Prognosis difficulties can affect reputation	“Discussion is difficult, because people behave very differently and people progress very differently. You know some people have terrible bony disease that causes so much pain. Most people end up just getting a severe infection and just dying of the infection. So yeah, I don’t. Yeah, I don’t know. I don’t. Yeah, wait. I wait till the end to kind of have those conversations.” *(Hematologist)*

### Growth despite a life-limiting diagnosis

Participants with MM and SMM described experiences of psychological growth despite the challenges of living with an incurable disease, through acceptance, considering life values and revaluation of priorities. Acknowledging the inevitability of death, some emphasized that life is unpredictable, underscoring the importance of living fully in the present. Spirituality also emerged as a pathway for comfort, with some participants drawing strength from faith or continuing bonds with deceased loved ones.

*… “I do believe very much so that there is somewhere else to go. Because of things that have happened when my mother died, and I feel like, well, if I’m going to go, I’m going to mum. You know what I mean, and I talk to her every day, anyway.” (Female, MM, 60–70 years)*

For others, the illness prompted self-reflection on their life journey, opportunities for learning, and a deepened appreciation of meaningful activities and relationships.

*… “I guess probably the most positive one is being able to share my experience with friends, you know. Who are, you know, suffering from? Have multiple myeloma and to being quite comfortable with the fact that you know it will come back, and I will die. And and I kind of, I’m okay with that.” (Male, MM, 70–80 years)*

### The lonely burden of dying

Despite the experiences of personal growth, participants described death as an unspoken and often avoided aspect of living with MM and SMM. For many, their diagnosis represented the first significant confrontation with their own mortality, prompting fears about the future, loved ones, and the finite nature of life. While some participants expressed a desire for more open conversations about death with family and friends, many also described suppressing these thoughts to protect themselves from emotional distress.

*…. “It’s just like a discipline of just don’t go there because there is no point, and it will just suck my energy and make me feel terrible. And at the start I couldn’t do that. I used to wake up in sweats in the nighttime. Going. ‘Oh, my God, I’m going to die! I’m going to leave my children. They’re not going to have a mother.’ And it was just chewing up mental space in my head and making me feel terrible. And so I’ve stopped doing that. It’s not worth it. (Female, SMM, 40–50 years)*

Yet, alongside avoidance, participants expressed a clear desire for death to be normalized as a part of life, emphasizing the value of open dialogue to reduce fear and stigma.

*….. “I’m just dealing with it. I’m just getting on with it. You know, I’m under no illusions of where it’s going to end up. But I was born terminal. So it’s you know, we’re all going to die of something somewhere. Someday it’s just. It only bothers me how it affects my family.” (Male, MM, 70–80 years)*

Participants highlighted the personal weight of preparing for death, encompassing both practical and interpersonal challenges. While some struggled with practical matters such as financial preparations, others found the silence or withdrawal of friends to be particularly painful, with avoidance reinforcing feelings of isolation.

*…. “But you know my wife keeps saying ‘you don’t need to worry about that stuff, we’ll manage’. But but that sort of plays on the in the back of my mind all the time, and we have just recently sought financial advice. But I don’t know where that’s gonna really lead us. But yeah, I that’s kind of the main concerns that I have like ‘what will happen if I’m not around’ and my income’s not around. (Male, SMM, 40–50 years)*

### Clinician reluctance to talk about death

Clinicians reported experiencing personal discomfort when discussing death and dying with patients, leading to avoidance or minimization of these conversations, likely compounding the experience of death as being the “elephant in the room.”

*…. “You can never predict, you know, can’t predict the course. I find it very difficult to predict how long people live. Sometimes I give people these dismal outlooks, and then, like a few months later, I would look like a fool because they’ve lived for so long.” (Hematologist)*

Clinicians were concerned about causing further distress, as prognosis may feel repetitive or an emotionally burdensome topic to revisit, coupled with perceptions (or stated preferences) that patients may not want to talk about dying.

*…. “Well, when you’re having a conversation with them, and then, as soon as you touch on a nerve of something that is triggering and death tend to be quite triggering for a lot of people. Then you can have unexpected responses.” (Myeloma Nurse)*

Some perceived these conversations as unhelpful or harmful, potentially increasing distress or diminishing hope for their patients.

*…. “it’s such a slippery slope for someone’s mental health. If you do start talking about it. You’ve got to be so, you can read it in an instant in their eyes. Yeah, that’s a tricky one. We do because you talk about it being incurable. But we hope it’s a chronic disease that you die with, not from. But every time you’re progressing. your side effects are going to get worse. Your disease control isn’t going to be as great generally.” (Myeloma Nurse)*

## Discussion

Taken together, these findings support Symons et al.’s [4] proposal that dimensions of flourishing possible even as life ends. Participants demonstrated capacity for growth, meaning-making, and spiritual connection despite a life-limiting diagnosis. However, significant barriers related to death avoidance and relational burden limited fuller expression of flourishing. Each theme is discussed in turn, exploring clinical, systemic, and cultural barriers to flourishing and opportunities for intervention.

The theme of “*growth despite a life-limiting diagnosis*” aligns with existing evidence that healthcare systems often prioritize physical symptoms at the expense of personal growth and meaning-making [14]. Available interventions such as dignity therapy or meaning-centered psychotherapy can be useful to support psychological growth, by helping patients document their life stories and legacy, enhancing purpose [15, 16]. Importantly, these solutions can be offered as a complement to treatment options.

The “*lonely burden of dying*”* highlights a key emotional barrier to flourishing*, with participants often minimizing their own fears to protect family or friends from discomfort. This dynamic underscores how the burden of dying is not only about managing one’s own mortality but also navigating the emotional needs of others, often leaving patients unsupported in their own processing of death.

*Participants’ efforts to protect family and friends from discomfort often left them isolated in processing mortality.* The perception of “being a burden” reflects both cultural avoidance of death and death anxiety [17], along with inadequate support for patients and families to navigate end-of-life dynamics, eroding aspects of flourishing such as dignity and connection. Multidisciplinary teams, including psychologists and social workers, can provide family-centered support and advance care planning, reducing isolation [18].

“*Clinician reluctance to discuss death*”* further compounds these challenges.* This reluctance, often for genuine and appropriate reasons (such as diminishing hope or discouraging viable treatment options), may limit opportunities for patients to process existential concerns and thwart flourishing. In an effort to protect families or shield patients from perceived emotional burden, information may be withheld, delaying open and honest dialogue about death [19]. While resources exist to support patients to process death within the context of terminal illness, clinicians may hesitate to introduce them, either from lack of time during clinical reviews, reluctance due to uncertain prognoses, or from concerns the perceived expectation that specialists should focus on prolonging life [20].

Early integration of palliative care principles can minimize these barriers to flourishing [4]. However, palliative care is often seen as a last resort and points to a friction between appropriate timing of referral to options such as palliative care and diminishing hope for patients and their families. This reluctance is driven by misconceptions equating palliative care with hospice care, as clinicians often associate it with terminal care [21]. Communication challenges, including fear of undermining patient morale and prognostic uncertainty in multiple myeloma, as expressed by interview participants, compound these barriers, alongside systemic issues like limited access to palliative care specialists and lack of standardized referral criteria [22]. This is especially compounded in multiple myeloma where a person may live with their terminal disease for years, and hence, “end of life” may *seem* distant [22].

## Conclusion

This study highlights the potential to promote flourishing among people living with multiple MM and SMM, despite their life-limiting diagnosis. Key findings underscore individual capacity for psychological growth, the need to remove barriers around conversations about death, and the emotional burden of dying in the context of cultural death anxiety. Although the clinical trajectories of MM and SMM differ, participants across both groups described remarkably similar fears related to mortality, disease progression, loss of independence, and the impact of their illness on loved ones. However, people living with SMM may face unique challenges, as they often experience prolonged uncertainty while receiving less frequent clinical contact and having reduced access to cancer-specific supportive care services [23]. This can leave individuals navigating significant psychological distress with fewer opportunities for professional support and intervention.

Healthcare systems could consider the integration of evidence-based interventions like dignity therapy and meaning-centered psychotherapy to support meaning-making and spiritual fulfillment [24]. Breaking the silence around death may involve early referral to palliative care and clinician training in existential communication to reduce stigma and align care with patient goals [25]. Building meaningful, respectful relationships between hematological and palliative care services may ensure that people with MM, and those with SMM who experience significant distress, have access to timely, responsive, and appropriate supportive and palliative care when it is likely to provide the most benefit for their ability to live as well as possible in the context of terminal illness. Further, multidisciplinary teams, including palliative care specialists, psychologists, and social workers, can alleviate the perceived burden of dying by addressing practical and interpersonal challenges through family-centered support and advance care planning.

## Supplementary Information

Below is the link to the electronic supplementary material.

ESM1 (19.0 KB DOCX)

## Data Availability

No datasets were generated or analysed during the current study.
