# Supplementary material for: Missed opportunities to promote flourishing in cancer care: a brief examination of multiple myeloma
Source: Support Care Cancer. 2026 Jul 8;34(8):746. doi: 10.1007/s00520-026-10978-3 (PMC13346150; doi:10.1007/s00520-026-10978-3)
Supplement: Supplementary file 1 — (19.0 KB DOCX) [file 520_2026_10978_MOESM1_ESM.docx]

**Supplementary Information: Missed opportunities to promote flourishing in cancer care**

**Patient Interview Questions**

1. Tell me about your experience with receiving care to manage your *physical* health*?*

*Prompt: How are you feeling about the care you are receiving?*

*Prompt: Did you feel these issues were adequate addressed?*

2. In an ideal world how would you like to experience care for your physical health?

*Prompt: By whom, where and in what context?*

3. Tell me about your experience in receiving care for your mental health care?

*Prompt: How are you feeling about the mental health care you are receiving?*

*Prompt: Did you feel these issues were adequate addressed?*

4. In an ideal world how would you like to experience care for your mental health?

*Prompt: By whom, where and in what context?*

*Prompt: Can you explain your different perception in the care for your physical and mental health?*

5. How are you managing with your diagnosis?

*Prompt: Where did you hear about these things?*

*Prompt: How effective are your strategies?*

*Prompt: Where would you like this information to be found or come from?*

*Prompt: Have there been any positive changes since being diagnosed with MM?*

6. Is there anything else you would like to add about the support you are receiving?

*Prompt: Social; spiritual; informational; emotional; practical aspects*

8. Do you have any further comments to add?

Prompts

- “Is anything important missing?”
- “Can you tell me more about that?”
- “What do you mean by that?”
- “Can you give me an example?”

**Health Professionals - Interview Questions**

1. What care do you provide to people living with MM?

*Prompt: public, private, metro, state/location*

*How is that different from people living with SMM and MGUS?*

1. From your perspective, what are your thoughts about the current level of supportive care that is provided to individuals with MM?

*Prompt:* supportive care framework prompts (social, emotional etc).

*Prompt: How is that different from people living with SMM and MGUS?*

*Prompt: What are the gaps in supportive care?*

*Prompt: What are the barriers to meeting supportive care needs?*

*Prompt: What are you thoughts on the care that is provided for their mental health?*

*Prompt: What are you thoughts on the care that is provided for their quality of life?*

1. What are your thoughts on comprehensive supportive care? (ie, emotional support, physical)

*Prompt: Where should this fit within the patient’s navigation of the health care system?*

1. In an ideal world, what would supportive care look like for people living with MM?

*Prompt: By whom, how?*

*Prompt: How would it be different for people with SMM and MGUS?*

Prompts

- “Is anything important missing?”
- “Can you tell me more about that?”
- “What do you mean by that?”
- “Can you give me an example?”
